# Supplementary figures and images for: Flexible resonance in prefrontal networks with strong feedback inhibition
Source: PLoS Comput Biol. 2018 Aug 9;14(8):e1006357. doi: 10.1371/journal.pcbi.1006357 (PMC6103521; doi:10.1371/journal.pcbi.1006357)

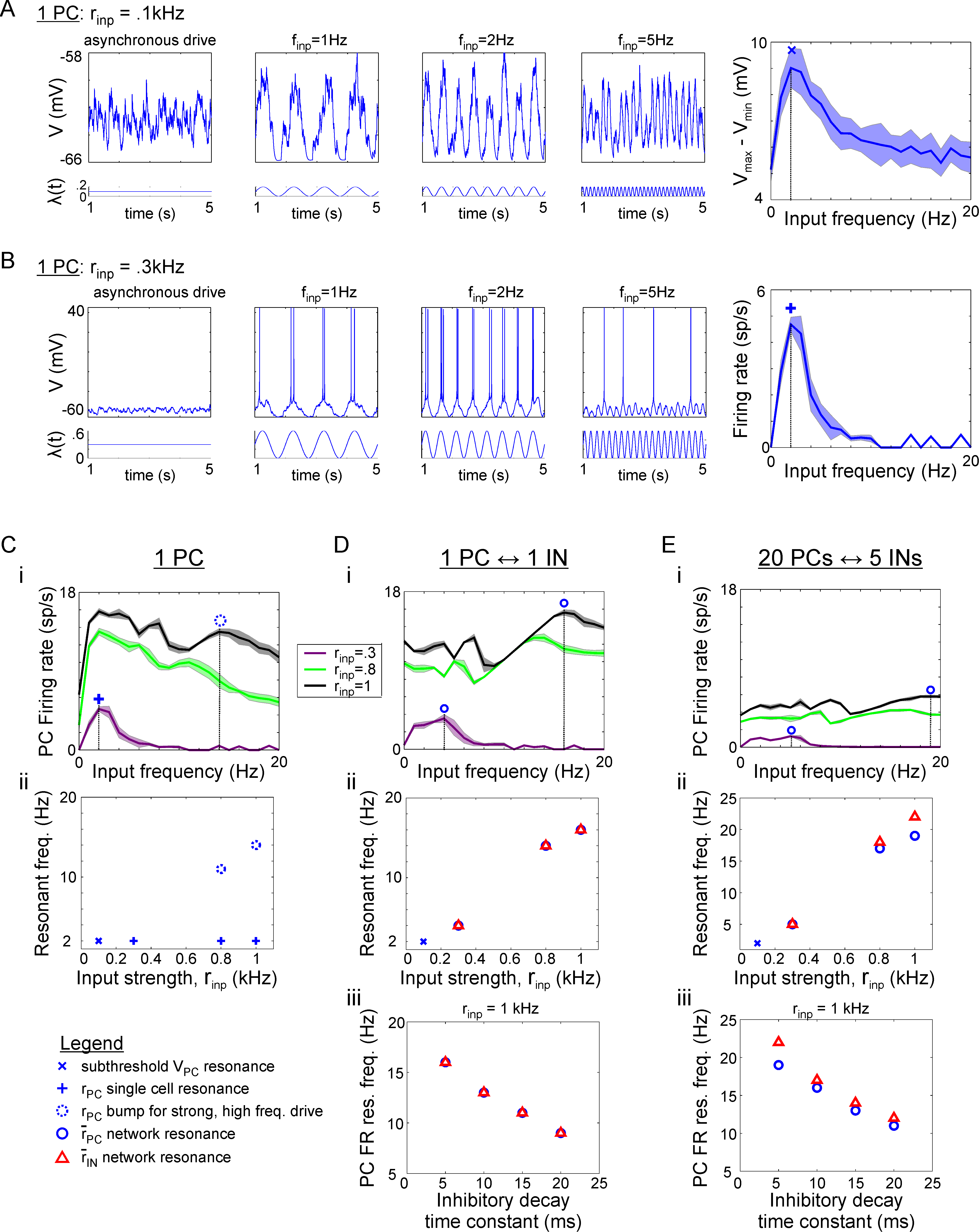

Supplement: S1 Fig — (A) After removing background noise to allow for subthreshold fluctuations, subthreshold resonance in the voltage fluctuation was observed at 2 Hz in a single principal cell (PC) driven by a weak sinusoidal drive (rinp = .1 kHz). Example voltage traces are shown in response to asynchronous input and sinusoidal inputs at f = 1 Hz, 2 Hz, and 5 Hz. The amplitude of voltage fluctuation, Vmax − Vmin is plotted versus input frequency, and the peak is marked with a × symbol. (B) After the input strength was increased to a slightly suprathreshold level (rinp = .3 kHz), suprathreshold spiking resonance was observed at the same 2 Hz frequency in a single PC. Example voltage traces are shown in response to asynchronous input and sinusoidal inputs at f = 1 Hz, 2 Hz, and 5 Hz. The time-averaged firing rate (FR) is plotted versus input frequency, and the peak is marked with a + symbol. This suggests that the subthreshold resonance translates to suprathreshold resonance in the linear regime. (C) (i) FR profile showing that as the strength of sinusoidal input is increased further to rinp = .8 kHz and 1 kHz, spiking resonance in the single PC remains at the same frequency and multiple bumps in time-averaged firing rate emerge at higher frequencies. The dotted circle marks the frequency at which an input strength-dependent bump in firing rate occurs (in the single PC) that is closest to the global maxima in the network. (ii) The scatter plot shows the continuity between the input strength-independent subthreshold and suprathreshold resonances in the single PC. (D) Response of a minimal PC/IN network with one PC and one IN. Strength of the PC → IN synapse was increased so that a spike in the single PC elicited a spike in the IN, and the background noise was removed for comparison with the resonant response of the single PC. (i) PC firing rate profiles in the minimal network given sinusoidal inputs with suprathreshold input strength rinp = .3, .8, 1 kHz. Feedback inhibition suppressed [file pcbi.1006357.s001.tif]

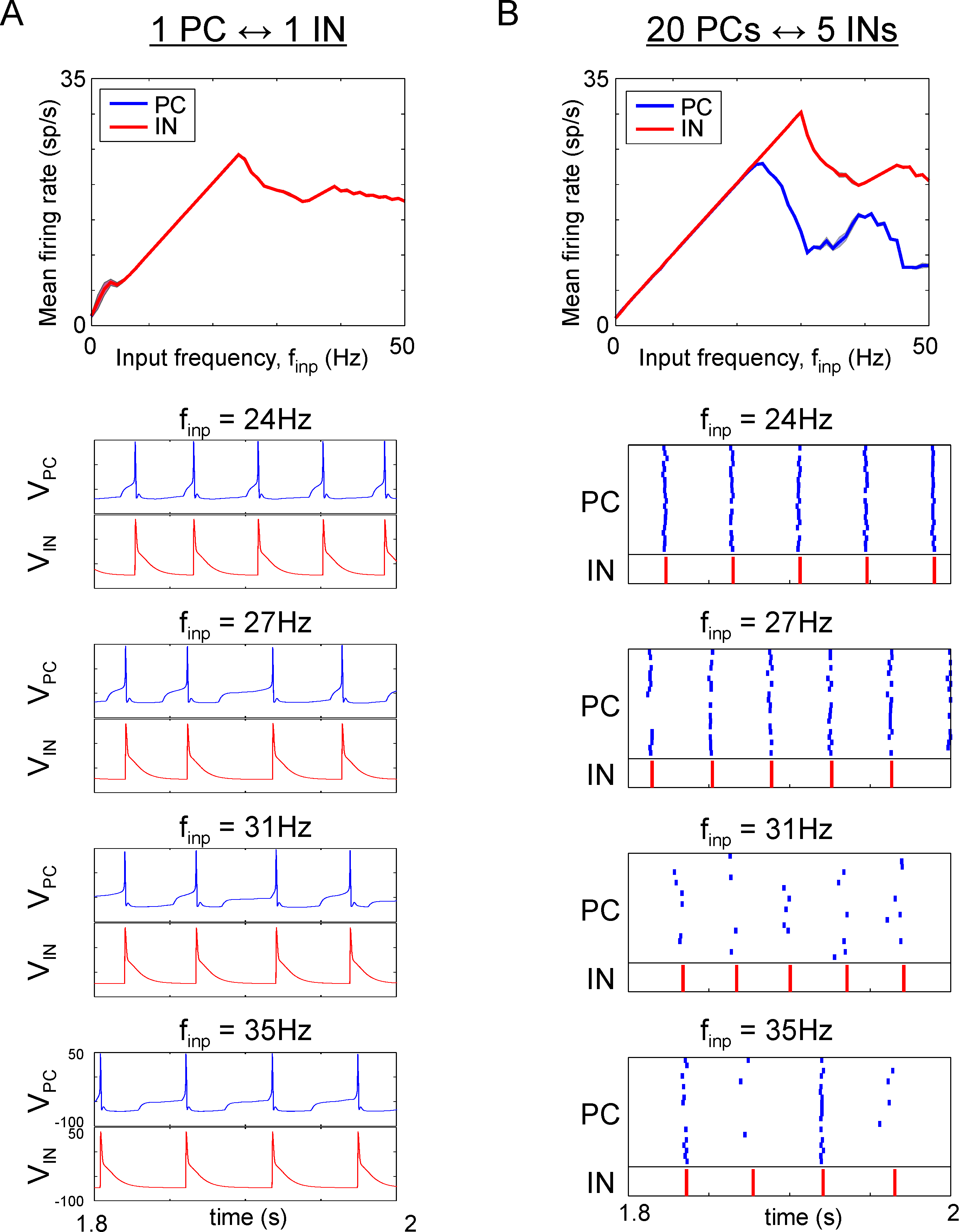

Supplement: S2 Fig — (A) Time-averaged firing rate profiles of PC (blue) and IN (red) cells in a minimal PC/IN network with one PC and one IN given high synchrony, square wave input. The IN can spike only when the PC spikes which causes their firing rates to peak in response to the same input frequency. (B) Time-averaged firing rate profiles of PC (blue) and IN (red) populations in the full PC/IN network given high synchrony, square wave input. The IN cells continue spiking after the firing peaks in the PC population because even a subset of PCs spiking on every cycle of the input is sufficient to engage all the INs. (TIF) [file pcbi.1006357.s002.tif]

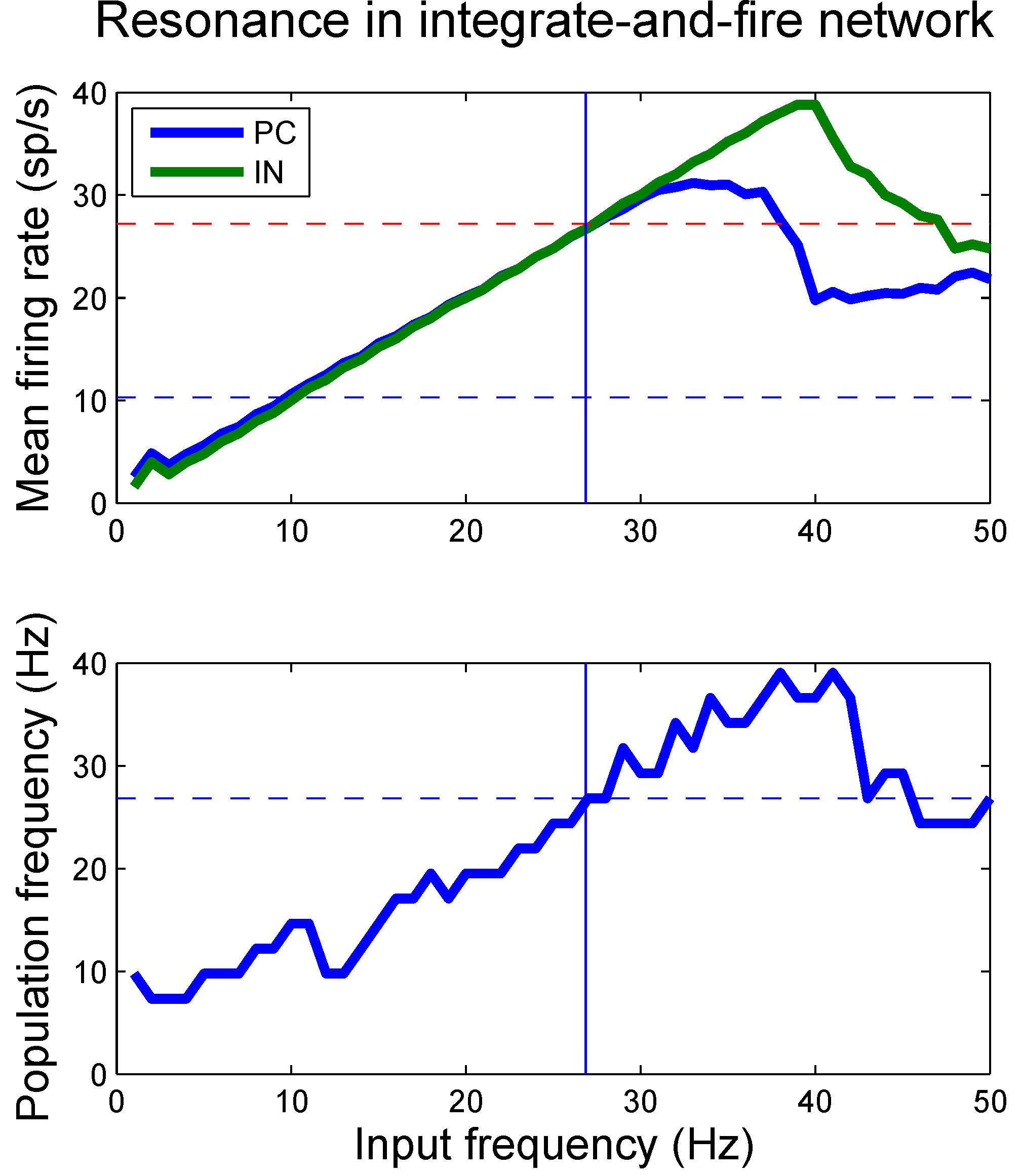

Supplement: S3 Fig — Qualitative features of the PFC network model were reproduced in a simpler PC/IN network model with leaky integrate-and-fire (LIF) neurons. (top) Firing rate profile for PC (blue) and IN (red) populations. (bottom) Population frequency profile for PC and IN populations. Peak population frequency occurs at the input frequency maximizing IN activity (i.e., feedback inhibition). LIF neurons were modeled with membrane potential V (mV) governed by: dVdt=-Iinp(t,V)-gl(V-El)-gsyn(V-Esyn) where t is time (ms), gl = 0.1, El = − 65 mV, Iinp(t, V) is an excitatory current (μA/cm2) reflecting inputs from external sources described in the Methods section, and Isyn denotes synaptic currents (μA/cm2) with double exponential conductances driven by other populations. When the membrane potential reaches the threshold of 0 mV, the voltage is reset and held at -65 mV for a refractory period of 3 ms. There were 25 PCs and 5 INs. For PCs, synaptic inputs were inhibitory with gsyn = 0.1, Esyn = −80 mV, 2 ms decay and 0.4 rise time constants. For INs, synaptic inputs were excitatory with gsyn = 0.03, Esyn = 0 mV, 10 ms decay and 0.2 rise time constants. Inputs to the LIF network were the same as the more detailed PFC network described in the Methods section except that ginp = .00375 mS/cm2 and gnoise = 0.0056 mS/cm2. (TIF) [file pcbi.1006357.s003.tif]

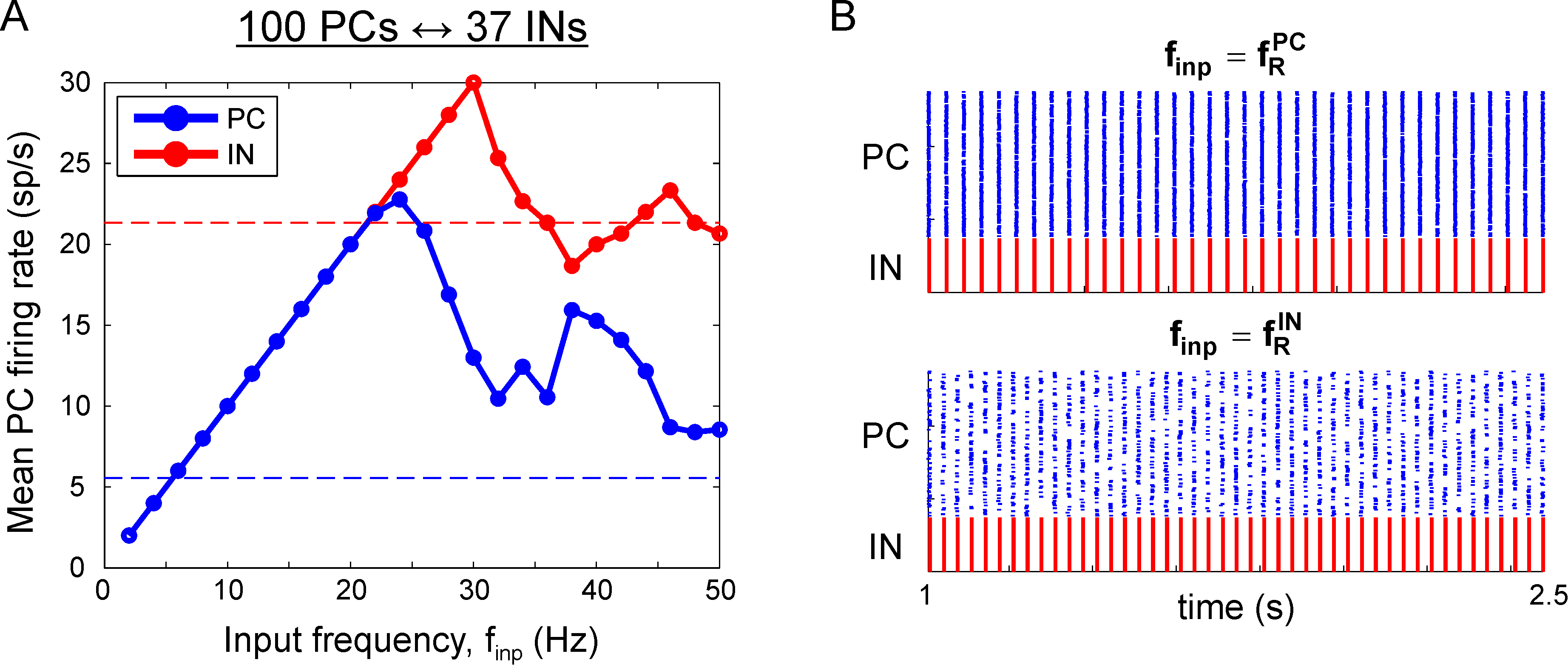

Supplement: S4 Fig — (A) Firing rate profile for PC (blue) and IN (red) populations in a PFC network with 100 PCs and 37 INs. All parameters of the model were kept fixed relative to the control model except the number of cells per population was increased. Note that the resonant frequencies are the same as in the control model with 20 PCs and 5 INs. (B) Raster plots showing maximal PC spiking at finp=fRPC and maximal IN spiking (with less PC spiking) at finp=fRIN. (TIF) [file pcbi.1006357.s004.tif]

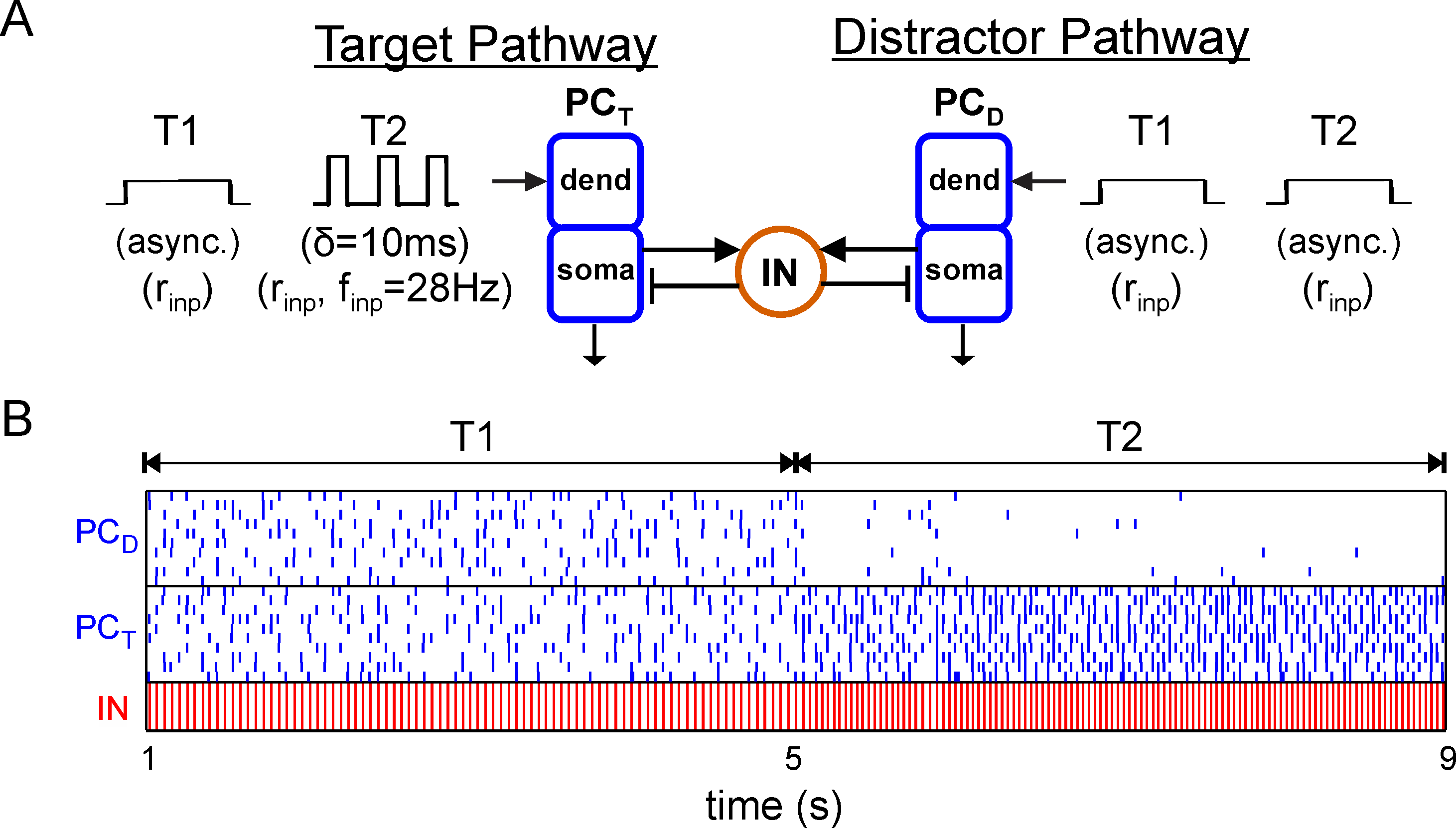

Supplement: S5 Fig — (A) Diagram showing a target PC population, PCT, driven by an asynchronous input during period T1 then a medium-synchrony oscillatory input at the 28 Hz fpop-resonant frequency during period T2 in competition with a distractor PC population, PCD, driven by an equal-mean asynchronous input during both periods. (B) Raster plot showing that no suppression of either population occurs when their population frequencies are the same during period T1 but that PCD is suppressed within a cycle of PCT oscillating more quickly during period T2. (TIF) [file pcbi.1006357.s005.tif]

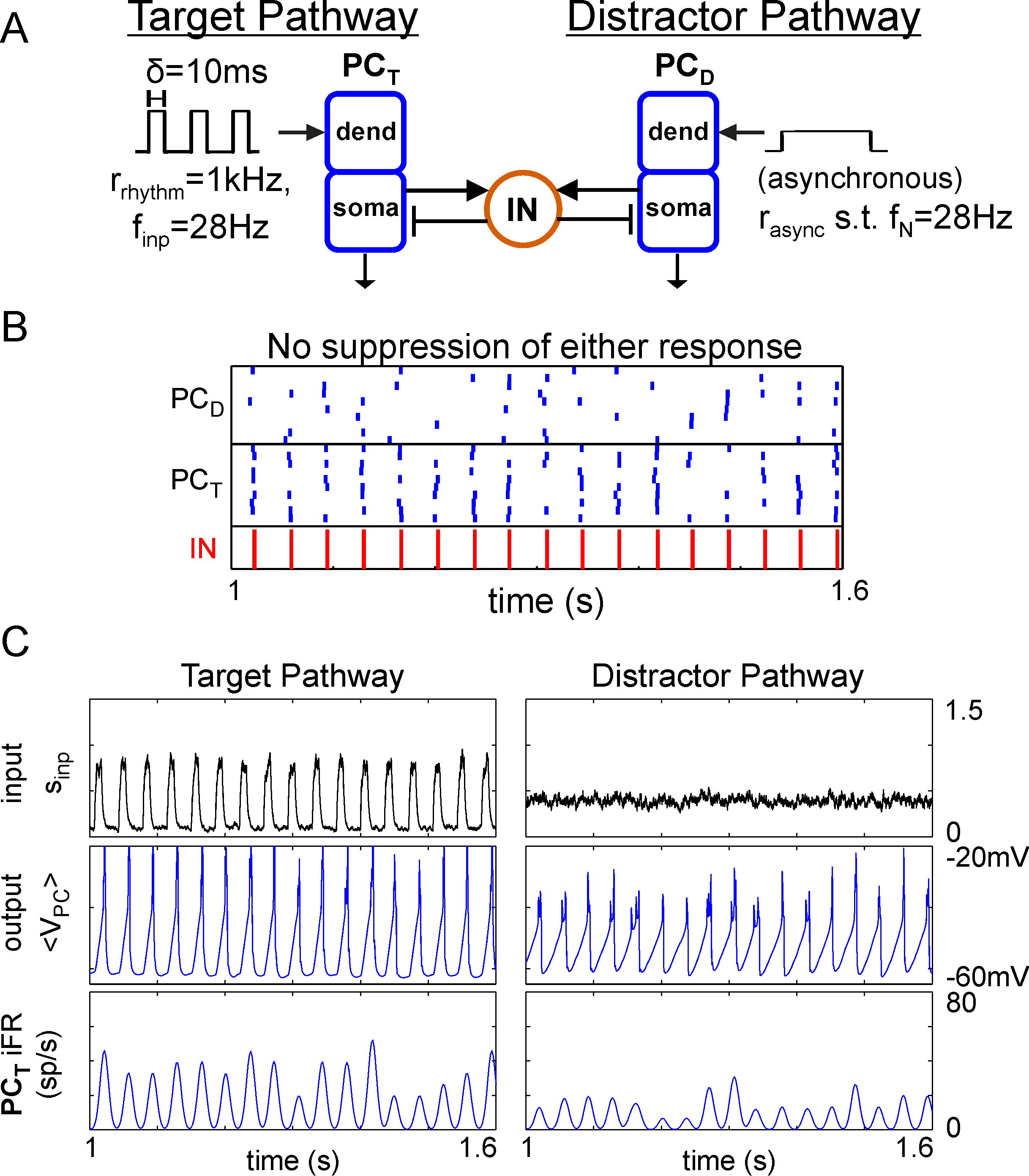

Supplement: S6 Fig — (A) Diagram showing a target PC population, PCT, driven by medium-synchrony oscillatory input at the 28 Hz fpop-resonant frequency in competition with a distractor PC population, PCD, driven by a higher-rate asynchronous input that produces a 28 Hz natural oscillation. (B) Raster plot showing that synchronous spiking occurs in both populations with time-averaged firing rates that would be expected in each population given their inputs in the absence of competition. (C) Plots showing the (top) input, (middle) mean population voltage, and (bottom) instantaneous firing rate for (left) PCT and (right) PCD populations. (TIF) [file pcbi.1006357.s006.tif]

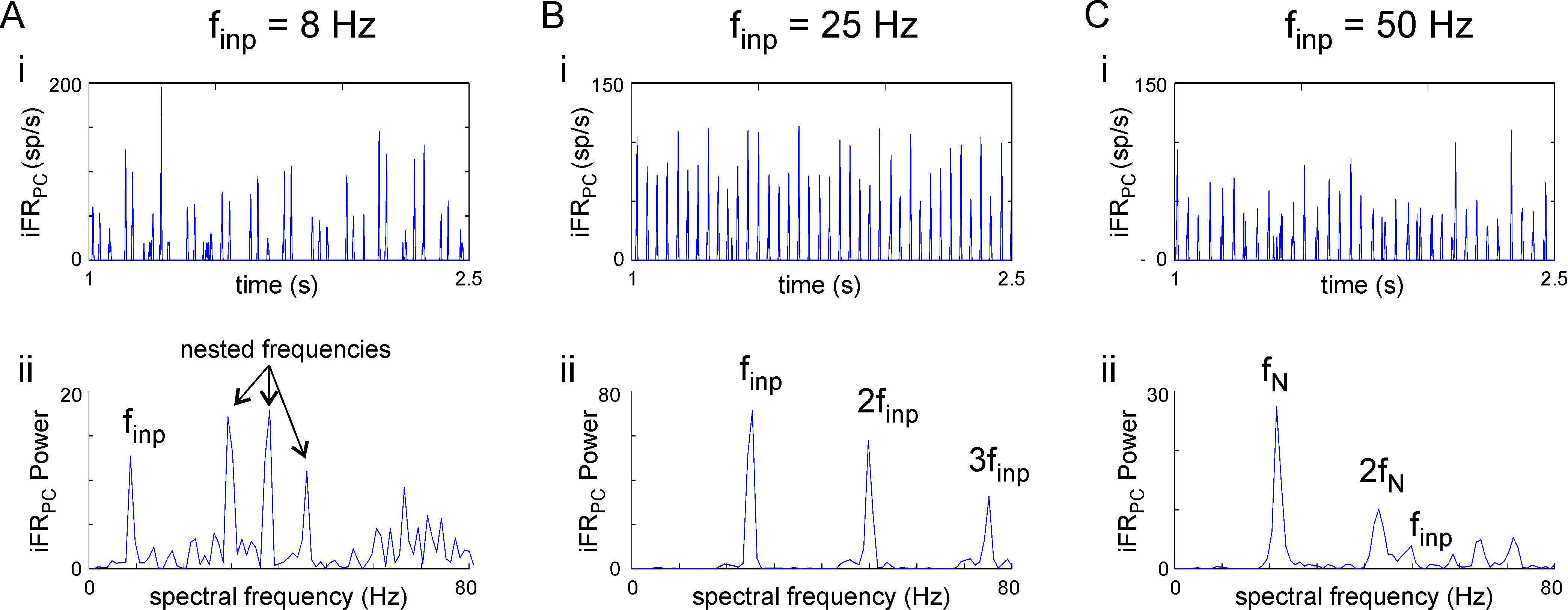

Supplement: S7 Fig — (A) Response to 8 Hz sinusoidal drive. (i) Nested oscillations in the PC iFR. (ii) Power spectrum with peaks at both the external driving frequency and frequency of internally-generated, nested oscillations. Low-synchrony square wave inputs can also produce nested oscillations. Across realizations, different frequencies may have peak power, which results in ambiguity when fpop is defined as the frequency with peak power. However, this does not affect the current study because nesting only occurs at frequencies well below the time-averaged firing rate peaks of ongoing inhibition-based oscillations investigated in this work. (B) Response to 25 Hz sinusoidal drive. (i) PC population with instantaneous firing rate locked to the period of the input; this occurs for intermediate frequencies of a sinusoidal input and all frequencies of a high-synchrony square wave input up to the IN firing rate resonant frequency. (ii) Power spectrum with peaks at the external driving frequency and its harmonics. (C) Response to 50 Hz sinusoidal drive. (i) PC population with instantaneous firing rate paced by the network’s internal time constants; this occurs for driving frequencies above the IN firing rate resonant frequency. (ii) Power spectrum with prominent peaks at the internally-generated, natural frequency and its harmonics as well as a much smaller peak at the external driving frequency. The response to 50 Hz square wave drive, independent of the degree of input synchrony, exhibits a similar asymptotic behavior. (TIF) [file pcbi.1006357.s007.tif]

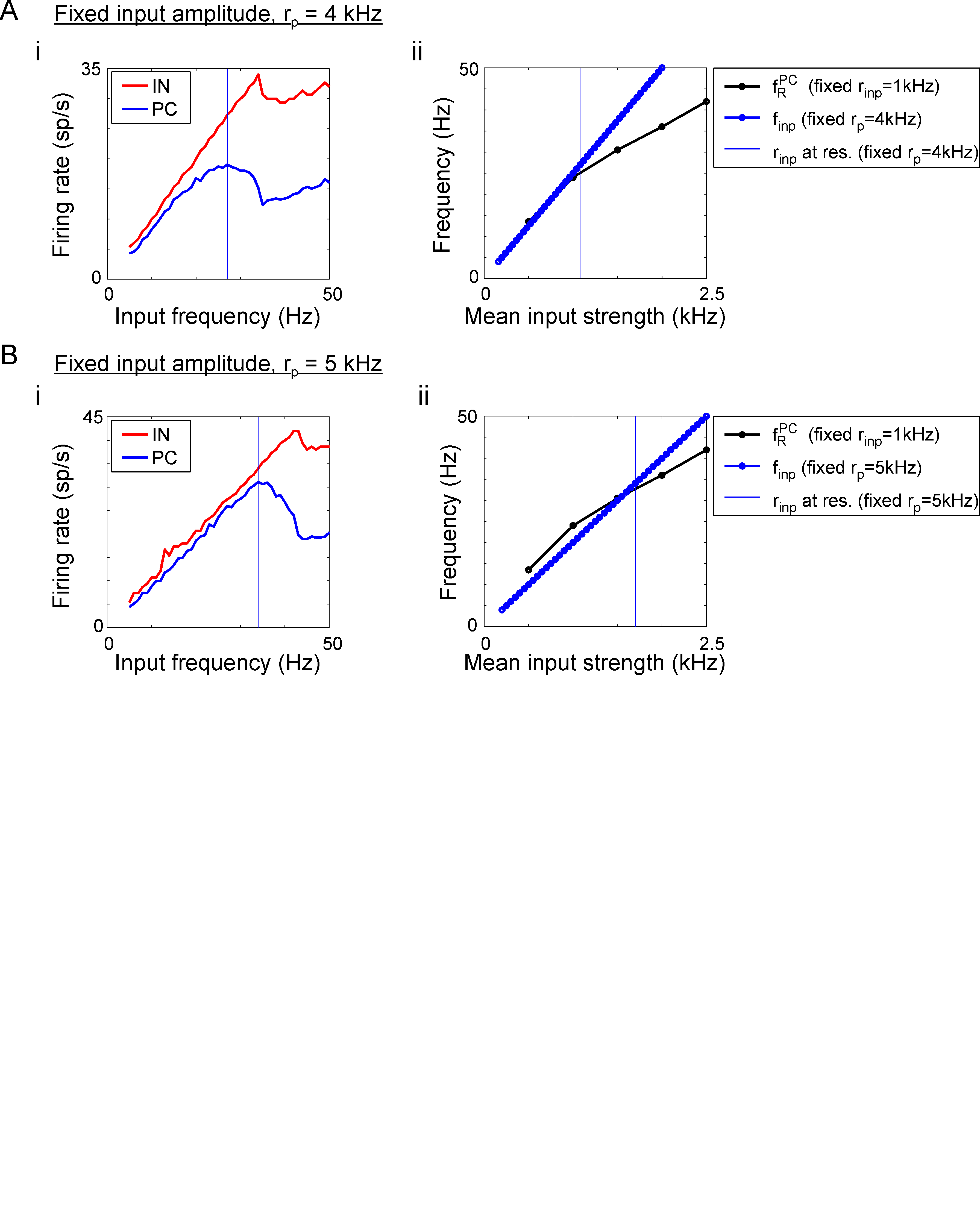

Supplement: S8 Fig — (A) Response to fixed-amplitude square wave with pulse amplitude fixed to rp = 4 kHz for all driving frequencies. (i) Firing rate (FR) profile for medium-synchrony square waves. Given fixed pulse amplitude, mean input strength increases with input frequency, proportionally to (pulse amplitude) × (inter-pulse frequency) × (pulse width), as an increasing number of 4 kHz pulses occur in the same period of time; 4 kHz corresponds to the amplitude of a fixed-mean square wave pulse when rinp = 1 kHz and finp = 25 Hz. FRs peak at higher frequencies than in response to a fixed-mean square wave. (ii) Plot showing (1) the resonant frequency of peak PC FR given fixed-mean square waves for different drive strengths, rinp (black), (2) the fixed-amplitude input frequencies corresponding to different drive strengths (blue), and (3) a vertical line marking the fixed-amplitude drive strength at the first peak of PC FR in (Ai). The intersection of these curves shows that the first peak in the fixed-amplitude FR profile in (Ai) occurs when the mean strength for a given finp establishes an input strength-dependent FR resonant frequency (determined using fixed-mean square waves) that matches the input frequency. (B) Same as (A) except the pulse amplitude was fixed to rp = 5 kHz for all driving frequencies. 5 kHz corresponds to the amplitude of a fixed-mean square wave pulse when rinp = 1 kHz and finp = 20 Hz. (i) Firing rates peak at an even higher input frequency. (ii) Compared to (Aii), the blue curve shifted to the right because every frequency is associated with a higher mean firing rate when the pulse amplitude increases. The frequency at which the peak occurs in (Bi) corresponds to the mean input strength marked with a vertical line. As in (A), the intersection of the three curves shows that the first peak in the fixed-amplitude FR profile in (Bi) occurs when the input frequency equals the FR resonant frequency given the mean input strength associated with a fixed-amplitude square [file pcbi.1006357.s008.tif]
